# Metabolite profiles and DNA methylation in metabolic syndrome: a two-sample, bidirectional Mendelian randomization

**DOI:** 10.3389/fgene.2023.1184661

**Published:** 2023-09-15

**Authors:** Alana C. Jones, Zsuzsanna Ament, Amit Patki, Ninad S. Chaudhary, Vinodh Srinivasasainagendra, Naruchorn Kijpaisalratana, Devin M. Absher, Hemant K. Tiwari, Donna K. Arnett, W. Taylor Kimberly, Marguerite R. Irvin

**Affiliations:** ^1^ Medical Scientist Training Program, University of Alabama at Birmingham, Birmingham, AL, United States; ^2^ Department of Epidemiology, University of Alabama at Birmingham, Birmingham, AL, United States; ^3^ Department of Neurology, Massachusetts General Hospital, Boston, MA, United States; ^4^ Department of Biostatistics, University of Alabama at Birmingham, Birmingham, AL, United States; ^5^ Department of Epidemiology, University of Texas Health Science Center, Houston, TX, United States; ^6^ Division of Neurology, Department of Medicine and Division of Academic Affairs, Faculty of Medicine, Chulalongkorn University, Bangkok, Thailand; ^7^ HudsonAlpha Institute for Biotechnology, Huntsville, AL, United States; ^8^ Office of the Provost, University of South Carolina, Columbia, SC, United States

**Keywords:** Mendelian randomization, metabolic syndrome, metabolomics, DNA methylation, multiomics

## Abstract

**Introduction:** Metabolic syndrome (MetS) increases the risk of cardiovascular disease and death. Previous ‘-omics’ studies have identified dysregulated serum metabolites and aberrant DNA methylation in the setting of MetS. However, the relationship between the metabolome and epigenome have not been elucidated. In this study, we identified serum metabolites associated with MetS and DNA methylation, and we conducted bidirectional Mendelian randomization (MR) to assess causal relationships between metabolites and methylation.

**Methods:** We leveraged metabolomic and genomic data from a national United States cohort of older adults (REGARDS), as well as metabolomic, epigenomic, and genomic data from a family-based study of hypertension (HyperGEN). We conducted metabolite profiling for MetS in REGARDS using weighted logistic regression models and validated them in HyperGEN. Validated metabolites were selected for methylation studies which fit linear mixed models between metabolites and six CpG sites previously linked to MetS. Statistically significant metabolite-CpG pairs were selected for two-sample, bidirectional MR.

**Results:** Forward MR indicated that glucose and serine metabolites were causal on CpG methylation near *CPT1A* [B(SE): −0.003 (0.002), *p* = 0.028 and B(SE): 0.029 (0.011), *p* = 0.030, respectively] and that serine metabolites were causal on *ABCG1* [B(SE): −0.008(0.003), *p* = 0.006] and *SREBF1* [B(SE): −0.009(0.004), *p* = 0.018] methylation*,* which suggested a protective effect of serine. Reverse MR showed a bidirectional relationship between cg06500161 (*ABCG1*) and serine [B(SE): −1.534 (0.668), *p* = 0.023].

**Discussion:** The metabolome may contribute to the relationship between MetS and epigenetic modifications.

## Introduction

Metabolic syndrome (MetS), defined by a cluster of cardiometabolic risk factors (hypertension, elevated blood glucose, abnormal lipid levels, and abdominal obesity), is described as “more than the sum of its parts” (Reilly and Rader, 2003; Huang, 2009). In the last 4 decades, the prevalence of MetS has risen by 35% overall, with consistent increases across all sociodemographic groups; further, more than one-third of all United States adults has MetS (Moore et al., 2017). MetS increases the risk of cardiovascular diseases more than the risk of these traits individually (Fahed et al., 2022). Previous metabolomics studies have discovered dysregulated serum metabolite concentrations in the setting of MetS, but mechanisms of how the metabolome confers MetS risk are unclear (Roberts et al., 2014; Azab et al., 2021; Carioca et al., 2021; Comte et al., 2021; Jialal et al., 2021; Wu et al., 2021; Zhang et al., 2021; Lind et al., 2022). Similarly, epigenome-wide association studies (EWAS) have identified, with consistent validation, alterations in DNA methylation of cytosine-phosphate-guanine (CpG) sites near genes that contribute to lipid metabolism, inflammation pathways, and hormone signaling in MetS. Importantly, the epigenome is sensitive to the environment (which may include the metabolome). Few studies have integrated the metabolome with MetS-related epigenomic sites, which could provide additional information on how the metabolome may alter individual risk for MetS.

Longitudinal data on ‘-omics’ are generally unavailable to understand causal relationships, particularly between -omic measurements (e.g., epigenomics and metabolomics). When only cross-sectional data are available, Mendelian randomization (MR) analyses provide an alternative. Importantly, past MR analyses have suggested that cardiometabolic traits are causal on methylation changes (Akinyemiju et al., 2018; Hidalgo et al., 2021; Jones et al., 2021). Also, metabolite reactions, such as the methionine cycle, may contribute to DNA methylation as methyl donors via DNA methyltransferase reactions (Zhang, 2018; Maddocks et al., 2016; Y et al., 2016). Yet, pathways of how MetS-related traits drive methylation variations have not been fully elucidated (Zaghlool et al., 2018). Methylation patterns associated with MetS that have been observed via EWAS could be downstream of dysregulated metabolite levels. Therefore, understanding the relationship between the metabolome and epigenome may help identify potential mechanisms of DNA methylation in cardiometabolic dysfunction. The objective of this study was to utilize genomic and metabolomic data from the Reasons for Geographic and Racial Differences in Stroke (REGARDS) Study, as well as genomic, epigenomic, and metabolomic data from the Hypertension Genetic Epidemiology Network (HyperGEN) to, first, conduct a multi-omics analysis of MetS and second, establish the directionality of the observed associations through bidirectional MR analyses.

## Materials and methods

### Study populations

We leveraged data from a subset of participants from the REGARDS Study. The REGARDS objectives and design have previously been described (Howard et al., 2005). Briefly, 30,239 community-dwelling, non-Hispanic Black and White adults aged 45 and older were recruited between 2003 and 2007 across the contiguous United States with oversampling from the Southeast region. Metabolomics were conducted in a subset of individuals who were part of a case-cohort study for incident strokes (N = 2,165) (Ament et al., 2022). A subset of these participants also had available genotype data (N = 1,865). Metabolite profiling, genotyping, and quality control metrics are detailed in the Supplementary Methods.

We also utilized available genomic (N = 1,898), epigenomic (N = 614), and metabolomic data (N = 300) from the Hypertension Genetic Epidemiology Network (HyperGEN). Recruitment methods for HyperGEN have been previously described (Williams et al., 2000). Briefly, HyperGEN—coordinated under the National Heart, Lung, and Blood Institute (NHLBI) Family Blood Pressure Program (FBPP)—was enriched for hypertensive adults and their siblings and/or adult offspring to evaluate genetic contributors to hypertension and target organ damage. Later, African American (AA) participants’ samples underwent deep coverage, whole genome sequencing (WGS) through the NLHBI Trans-Omics in Precision Medicine (TOPMed) Consortium. In separate ancillary studies, AA participant samples were selected for epigenotyping and metabolite measurements for studies of left ventricular hypertrophy (under an extreme phenotype design) and chronic kidney disease, respectively. Complete descriptions of data collection in HyperGEN are available in the Supplementary Methods. The different datasets used for this analysis are summarized in Supplementary Figure S1.

### MetS phenotyping

MetS was defined as having at least 3 of the following at the baseline visit according the United States National Cholesterol Education Program Adult Treatment Panel III (NCEP-ATP III) 2005 criteria: hypertension (SBP ≥ 130 mmHg or DBP ≥ 85 or self-reported use of anti-hypertensive medications); hypertriglyceridemia (triglyceride ≥ 150 mg/dL or self-reported use of lipid-lowering medication); hypoalphalipoproteinemia (HDL cholesterol <40 mg/dL for males or <50 mg/dL for females or self-reported use of lipid-lowering medication); elevated blood sugar (fasting blood glucose ≥ 100 mg/dL) or self-reported use of glucose-lowering medication or insulin); and abdominal obesity (waist circumference >40 cm for males or >35 cm for females (Huang, 2009).

### Statistical analyses

#### MetS-metabolite association and validation

**FIGURE 1:**
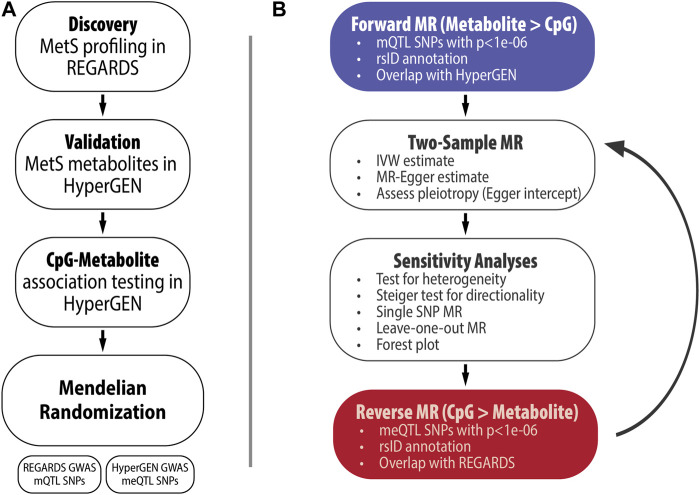
**(A)** Study Overview, **(B)** Summary of MR analyses.

All analyses are summarized in Figure 1. In the REGARDS case-cohort (N = 2,039), weighted logistic regression models were fit to test the association between prevalent MetS (outcome) and a panel of 162 serum metabolites (exposure). Models were adjusted for age, gender, race, cigarette smoking, alcohol use, and stroke case status. In sensitivity analyses, we also adjusted models for fasting status and restricted the analysis to the randomly selected controls (N = 872). Metabolites with association *p*-values less than the Bonferroni-corrected significance threshold of *p* = 0.05/162 = 3.09e-04 were selected for validation in HyperGEN participants with metabolomics data (N = 300). We also selected 12 metabolites that were upstream of DNA methylation pathways irrespective of statistical significance in REGARDS for validation. HyperGEN metabolite models were similarly adjusted for age, gender, smoking status, alcohol use, and recruitment center. Race was not included as a covariate, as all HyperGEN metabolomics participants were AA. Validated MetS metabolites (*p* <0.05) were selected for further analyses among HyperGEN participants who also had both metabolomic and methylation data (N = 134); none of these individuals were related. Logistic regression modeling was completed in SAS version 9.4.

#### Methylation studies

We evaluated the relationship between HyperGEN-validated metabolites and six CpG sites that have previously been linked to MetS (Table 1): cg06500161, annotated to ATP-Binding Cassette Subfamily G Member 1 (*ABCG1*); cg00574958, annotated to Carnitine Palmitoyltransferase 1A (*CPT1A*); cg02650017, annotated to Phosphoethanolamine/Phosphocholine Phosphatase 1 (*PHOSPHO1*); cg11024682, annotated to Sterol Regulatory Element-Binding Protein 1 (*SREBF1*); cg18181703, annotated to Suppressor Of Cytokine Signaling 3 (*SOCS3*); and cg19693031, annotated to Thioredoxin-Interacting Protein (*TXNIP*) (Hidalgo et al., 2021). We fit linear mixed models for the CpG beta score (outcome) and metabolite (predictor), adjusted for age, gender, recruitment center, left ventricular mass index, principal components of ancestry (PCs), Houseman-estimated cell counts, and family relatedness (random effect). For the linear mixed models, we used the *lme4* package in R (version 4.2.0). CpG-metabolite pairs with association *p* <0.05 were eligible for MR studies.

**TABLE 1:** CpG sites associated with MetS and related traits in the literature.

CpG	Gene	Location	Chr:BP	Function	MetS direction
cg06500161	*ABCG1*	shore	21:43,656,587	lipid transport	+
cg00574958	*CPT1A*	shore	11:68,607,622	fatty acid oxidation	-
cg02650017	*PHOSPHO1*	island	17:47,301,614	lipid biosynthesis and metabolism	-
cg18181703	*SOCS3*	shore	17:76,354,621	regulates cytokine activation	-
cg11024682	*SREBF1*	shelf	17:17,730,094	lipid metabolism and homeostasis	+
cg19693031	*TXNIP*	3′ UTR	1:145,441,552	mediates oxidative stress	-

Chr: chromosome, UTR: untranslated region. BP: base pair location corresponds to build GRCh37/hg19. ‘+’ indicates that increasing methylation at the CpG site is associated with a higher odds of MetS, whereas ‘-’ denotes that decreasing methylation at the CpG sites is associated with a higher odds of MetS in Hidalgo et al. (Hidalgo et al., 2021).

#### Two-sample, bidirectional Mendelian randomization

**FIGURE 2:**
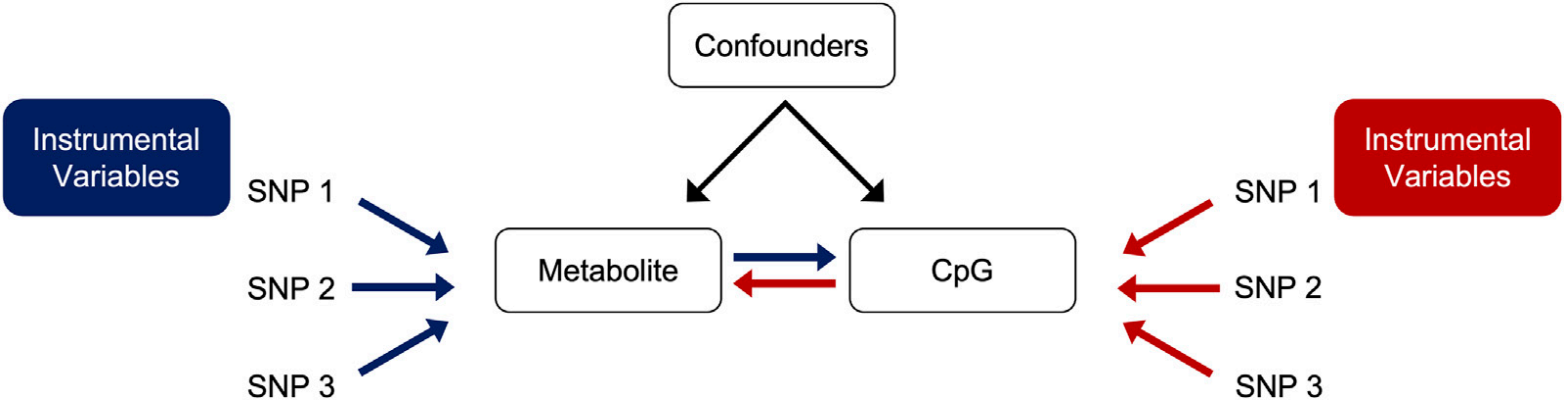
Directed acyclic graph (DAG) of forward (blue) and reverse (red) MR.

We conducted bidirectional MR using the *TwoSampleMR* package (version 0.5.6) in R (version 4.2.0) for statistically significant CpG-metabolite associations (Hemani et al., 2018). We hypothesized that metabolite levels were causal on CpG methylation (Figure 2). This MR approach uses single nucleotide polymorphisms (SNPs) associated with the trait of interest as the instrumental variables (IVs). Under the assumptions of the MR, if the SNPs that are strongly associated with the exposure are also associated with the outcome, then there is evidence to suggest a causal relationship. To obtain the exposure instruments (metabolite quantitative trait loci SNPs or mQTL SNPs), we conducted genome-wide association studies (GWAS) in PLINK 2.0 among participants with both metabolite and genetic data in REGARDS for selected metabolites, adjusted for age, sex, and the first ten PCs. We similarly conducted GWAS for selected CpG sites and corresponding WGS data in HyperGEN (N = 557) to obtain the outcome instruments (methylation quantitative loci SNPs or meQTL SNPs). HyperGEN meQTL models were adjusted for left ventricular mass index. Because the HyperGEN sample was all African American, we restricted the REGARDS GWAS to African American participants (N = 846) for SNP IV selection.

There were few SNPs in REGARDS with association *p* <5.00e-08, which corresponds to the traditional Bonferroni-corrected threshold for GWAS; thus, we considered a lower *p*-value threshold in order to increase the available IVs for forward MR analyses. Summary statistics for SNPs with association *p* <1.00e-06 in REGARDS metabolite GWAS (exposure IVs) and their corresponding estimates in HyperGEN (outcome IVs) served as the inputs for the MR in *TwoSampleMR*. We then harmonized allele orientation and effect estimates across the two cohorts. For CpG-metabolite pairs in which there was evidence to suggest a causal relationship in the forward direction (metabolite causal on the CpG, MR *p* <0.05), we investigated reverse causation as well. In reverse MR, the exposure IVs were SNPs that were strongly associated with the respective CpG in HyperGEN with *p* <1.00e-06 (methylation quantitative trait loci SNPs or meQTL SNPs), and the corresponding SNP associations from the metabolite GWAS were the outcome IVs.

We obtained MR estimates via multiple methods: Egger, weighted median, inverse variance-weighted (IVW), simple, and weighted. Generally, the IVW method provides the greatest statistical power, but it assumes balanced pleiotropy. Thus, when there was significant pleiotropy (Egger regression intercept *p* <0.05), we assessed the Egger estimate; otherwise, we considered the IVW estimate. We also conducted multiple sensitivity analyses for each CpG-metabolite pair in both forward and reverse MR (Burgess et al., 2017). These included tests for heterogeneity, single SNP MR (which computes MR estimates using each SNP as an individual IV instead of a combined effect of all the SNPs selected as IVs), and leave-one-out analyses—which computes MR estimates multiple times while leaving out each SNP from the set of exposure IVs to determine whether an individual SNP is the primary driver of the observed effect. We also conducted the Steiger test for causal direction, which determined whether the proportion of the variance in the exposure explained by the exposure IVs was significantly greater than the proportion of the variance in the outcome explained by those same IVs. In all analyses, two-sided *p*-values are assessed.

## Results

Baseline characteristics of the REGARDS and HyperGEN sub-study cohorts are presented in Table 2. In discovery models, 28 metabolites were significantly associated with prevalent MetS in REGARDS after correcting for multiple comparisons (Supplementary Figure S2). Of these, two metabolites (cystathionine and S-adenosylhomocysteine (SAH)) were known to be upstream of DNA methylation pathways. Of the 10 remaining methylation-related metabolites, the following were marginally associated with MetS (*p* <0.05): cystine, S-adenosylmethionine (SAM), sarcosine, serine. Unlike other methylation metabolites, higher serine levels were associated with a lower odds of MetS in REGARDS.

**TABLE 2:** Baseline characteristics of REGARDS and HyperGEN participants with metabolite measurements.

Trait N (%)/Mean (SD)	REGARDS (*n* = 2,039)	HyperGEN (*n* = 300)
Age	68.2 (10.4)	54.5 (9.5)
Gender, Male	1033 (50.7%)	124 (41.3%)
Race, Black	894 (43.8%)	300 (100%)
Current Smoker	306 (15.0%)	92 (30.7%)
Alcohol Use, None	1329 (65.2%)	221 (73.7%)
Prevalent MetS	1451 (71.2%)	215 (71.7%)

Moreover, associations were robust when we restricted the analysis to the controls in the stroke case-cohort and when we accounted for fasting status (Supplementary Table S1). Of the 38 total metabolites that were either statistically significant and/or are involved in DNA methylation pathways, 31 metabolites were available for analysis in HyperGEN. In validation models, D-gluconic acid (DGA), glucose, isoleucine, and leucine were strongly associated with MetS (*p* <1.00e-05). Additionally, 2-aminoadipic acid (2-AAA), alanine, glutamate, serine, and taurine were modestly associated with MetS (*p* <0.05). Associations for MetS metabolites in REGARDS and HyperGEN are presented in Supplementary Table S2.

In HyperGEN, 44.7% of participants in the metabolomics cohort also had available methylation data. The prevalence of MetS in this subset (74.6%) was concordant with the full metabolomics cohort (71.7%). In the methylation analysis, we assessed the relationships between the 9 validated MetS metabolites and the selected CpG sites. In the fully adjusted models, DGA, glucose, glutamate, isoleucine, serine, and taurine were associated with methylation of at least one CpG site. However, 2-AAA, alanine, and leucine, were not associated with any CpG site. Furthermore, cg18181703 (*SOCS3*) was not associated with any metabolite.

CpG-metabolite pairs that were statistically significant in linear mixed modeling were eligible for MR analysis (Table 3). All CpG-metabolite associations are presented in Supplementary Table S3. Of the 13 statistically significant CpG-metabolite associations, only glucose (*CPT1A*) and serine (*ABCG1*, *CPT1A*, *SREBF1*) metabolites showed causal effects on methylation in the forward MR (Table 4). Glucose SNPs were inversely associated methylation of cg00574958 (*CPT1A*). And serine-associated SNPs were inversely associated with methylation of CpG sites at *ABCG1* and *SREBF1*, though positively associated with CpG sites at *CPT1A* and *TXNIP*.

**TABLE 3:** CpG-metabolite associations in HyperGEN.

Metabolite	cg06500161 (*ABCG1*)		cg00574958 (*CPT1A*)		cg11024682 (*SREBF1*)		cg02650017 (*PHOSPHO1*)		cg19693031 (*TXNIP*)	
	B(SE)	*p*	B(SE)	*p*	B(SE)	*p*	B(SE)	*p*	B(SE)	*p*
DGA	--	--	--	--	--	--	−0.003 (0.002)	0.047	−0.017 (0.007)	0.020
Glucose	0.007 (0.003)	0.033	−0.007 (0.002)	0.005	0.012 (0.004)	0.003	--	--	−0.033 (0.007)	5.05e-06
Glutamate	−0.007 (0.003)	0.039	--	--	0.009 (0.004)	0.034	--	--	--	--
Isoleucine	--	--	--	--	--	--	--	--	−0.019 (0.008)	0.016
Serine	−0.009 (0.003)	0.002	0.007 (0.002)	0.002	−0.015 (0.004)	6.64e-05	--	--	--	--
Taurine	--	--	--	--	--	--	--	--	0.030 (0.007)	1.43e-05

Beta estimates (B) and standard errors (SE) presented for CpG-metabolite associations with *p* <0.05. ‘--' denotes that CpG-metabolite pair were not significantly associated (*p* ≥0.05). Linear mixed regression models were adjusted for age, gender, recruitment center, left ventricular mass index, principal components of ancestry, Houseman-estimated immune cell counts, and family relatedness (random effect).

**TABLE 4:** Summary of forward MR of metabolite levels on CpG methylation.

Metabolite	CpG (gene)	Forward MR				Sensitivity analyses			
		Method	n SNPs	B(SE)	*p*	Pleiotropy *p*	Heterogeneity *p*	Steiger test	
								*R* ^2^ metabolite	*R* ^2^ CpG
DGA	cg02650017 (*PHOSPHO1*)	IVW	7	−0.003 (0.002)	0.12	0.21	0.90	0.152	0.008
		Egger		−0.018 (0.011)	0.15				
	cg19693031 (*TXNIP*)	IVW		0.005 (0.006)	0.43	0.67	0.97		0.003
		Egger		−0.011 (0.036)	0.77				
Glucose	cg06500161 (*ABCG1*)	IVW	21	0.001 (0.002)	0.71	0.14	0.99	0.679	0.024
		Egger		−0.008 (0.006)	0.19				
	**cg00574958 (*CPT1A*)**	**IVW**		**−0.003(0.002)**	**0.028**	0.25	0.81		0.049
		Egger		−0.009 (0.005)	0.08				
	cg11024682 (*SREBF1*)	IVW		0.002 (0.002)	0.32	0.11	0.73		0.038
		Egger		0.014 (0.007)	0.07				
	cg19693031 (*TXNIP*)	IVW		0.005 (0.004)	0.18	0.91	0.70		0.048
		Egger		0.004 (0.011)	0.76				
Glutamate	cg06500161 (*ABCG1*)	IVW	4	0.001 (0.005)	0.83	0.73	0.94	0.083	0.001
		Egger		0.004 (0.008)	0.70				
	cg11024682 (*SREBF1*)	IVW		0.004 (0.006)	0.55	0.27	0.34		0.007
		Egger		0.016 (0.010)	0.25				
Isoleucine	cg19693031 (*TXNIP*)	IVW	8	0.006 (0.006)	0.33	0.98	1.00	0.171	0.003
		Egger		0.006 (0.017)	0.72				
Serine	**cg06500161 (*ABCG1*)**	**IVW**	9	**−0.008(0.003)**	**0.006**	0.28	0.55	0.227	0.028
		Egger		−0.024 (0.014)	0.13				
	**cg00574958 (*CPT1A*)**	IVW		−0.001 (0.003)	0.62	**0.022**	0.91		0.024
		**Egger**		**0.029(0.011)**	**0.030**				
	**cg11024682 (*SREBF1*)**	**IVW**		**−0.009(0.004)**	**0.018**	0.39	0.87		0.022
		Egger		0.006 (0.017)	0.72				
Taurine	cg19693031 (*TXNIP*)	IVW	17	−0.001 (0.004)	0.72	0.91	1.00	0.442	0.007
		Egger		−0.004 (0.018)	0.85				

IVW: inverse variance weighted; SNP: single nucleotide polymorphism. Boldface indicates statistical significance (*p* <0.05).

**FIGURE 3:**
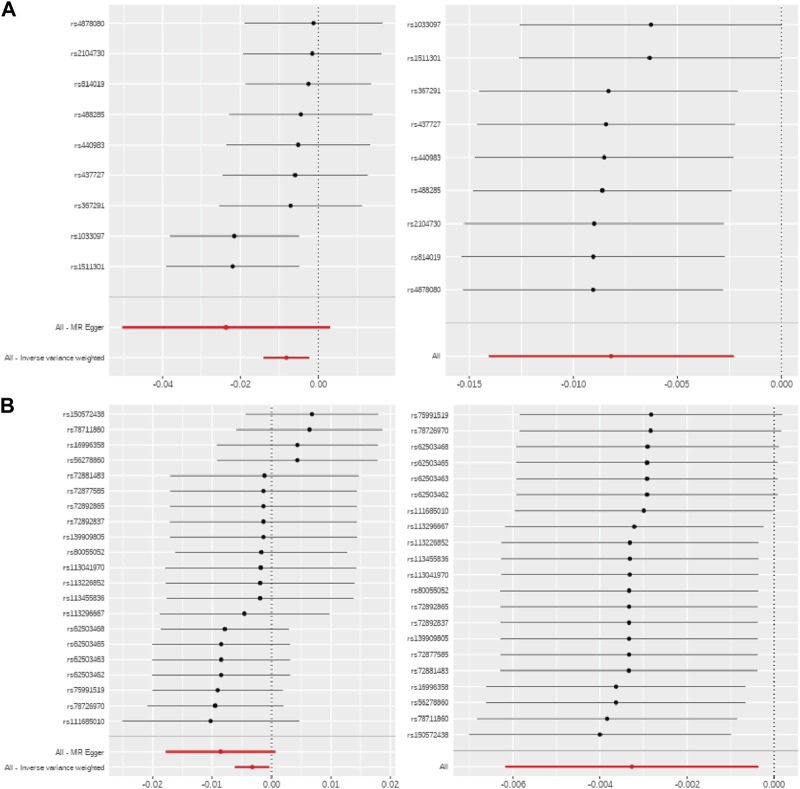
MR sensitivity analyses for serine and glucose metabolites. Single SNP MR (left) and leave-one-out MR (right) for **(A)** serine-*ABCG1* and **(B)** glucose-*CPT1A*.

Metabolite effects on methylation were robust in sensitivity analyses for the selection of SNPs as IVs, including leave-one-out analyses and single SNP MR (Figure 3). Moreover, tests for heterogeneity were not statistically significant for any CpG-metabolite pair, indicating that the SNPs that were used as IVs did not violate MR assumptions. And the Steiger tests confirmed the causal directionality of metabolite influence on methylation.

For metabolites with significant causal effects on methylation in the forward MR, reverse MR showed that CpG methylation was not causal on metabolites, apart from cg06500161 (*ABCG1*) on serine (B [SE]: −1.534 [0.668], *p* = 0.023). Results were consistent in leave-one-out and single SNP MR estimations. Because there were more than 700 IVs for *ABCG1* in reverse MR, we considered a *p*-value threshold of *p* <5.00e-08 to reduce the number of SNPs that were potentially in linkage disequilibrium (LD) with causal IVs. Complete reverse MR results are presented in Supplementary Table S4.

## Discussion

In this study, we leveraged individual-level metabolomic, epigenomic, and genomic data to, first, identify metabolites associated with MetS and, second, evaluate the relationship between these metabolites and CpG sites that have been linked to MetS in prior studies. We determined that serine and glucose may causally affect DNA methylation and that there may be a bidirectional relationship between serine and methylation of the *ABCG1* gene region. Notably, serine is also upstream of DNA methyltransferase reactions, unlike the metabolites (e.g., DGA) that were strongly associated with MetS and methylation but were null in MR analyses.

Previous metabolome-wide association studies (MWAS) for MetS have identified metabolites of statistical significance (Roberts et al., 2020; Azab et al., 2021; Carioca et al., 2021; Sun et al., 2021; Lind et al., 2022). However, replication of these metabolites across cohorts is lacking, and integration of additional ‘-omics’ data has been recommended to further explicate the pathophysiology of these variations in metabolite levels (Monnerie et al., 2020; Comte et al., 2021). For example, an integrative proteomics-metabolomics analysis identified metabolic pathways that were perturbed in the setting of MetS, including serine metabolism (Chen et al., 2022). Another study that combined metabolomic, proteomic, and transcriptomic data showed that mitochondrial respiratory chain dysfunction alters serine biosynthesis pathways (Bao et al., 2016). Although mechanisms are not fully understood, multi-omics approaches may illuminate biochemical pathways that contribute to MetS.

In this analysis, we detected causal relationships between serine and methylation of multiple MetS CpG sites. The inverse associations with CpG sites at *ABCG1* and *SREBF1*, as well as positive associations cg00574958 at *CPT1A,* suggest a protective relationship (e.g., increased *CPT1A* methylation is associated with less risk for MetS). Serine contributes to epigenetic modifications, as its metabolism supports the methionine cycle and subsequent methylation of DNA, RNA, and histones via methyltransferase reactions (Kadayifci et al., 2018; Gadecka and Bielak-Zmijewska, 2019; Lin et al., 2022; Pardo et al., 2022). The effect of serine metabolism on epigenetic variations has primarily been weighed in the context of cancer, but this metabolite could similarly affect cardiometabolic health via these same methylation pathways. For example, animal studies suggest that dietary supplementation with serine and taurine may prevent MetS (Ji et al., 2016; Rafiee et al., 2022; Wang et al., 2022). And a recent study in REGARDS found that serine levels were positively associated with a plant-based dietary pattern (Bhave et al., 2022). While mechanistic studies linking serine to DNA methylation and cardiometabolic dysfunction are lacking, current evidence provides a theoretical framework for the observed associations in this study.

EWAS for MetS and related traits have shown, with consistent validation across diverse populations, associations with the CpG sites we selected for this analysis (Akinyemiju et al., 2018; Hidalgo et al., 2021). Although this study was not able to link methylation to gene expression, previous studies have demonstrated this, as well as identified causal relationships with cardiometabolic traits in Mendelian randomization analyses (Cameron et al., 2023). Previously, Hidalgo et al. demonstrated that the selected CpG sites could be combined to form a methylation risk score for MetS in the HyperGEN (African ancestry) and GOLDN (European ancestry) cohorts (Hidalgo et al., 2021). Further, across multiple studies, cg00574958 (*CPT1A*) has demonstrated inverse associations with triglycerides and very low-density lipoprotein (VLDL) cholesterol (Irvin et al., 2014; Lai et al., 2016; Braun et al., 2017; Zheng et al., 2022). Similarly, inverse relationships between cg19693031 (*TXNIP*) and diabetes have been observed (Nuotio et al., 2020; Miller et al., 2023). These sites are located at or near CpG islands and other regulatory regions (Table 1). Thus, variations in their methylation may be linked to altered expression of genes that contribute to lipid metabolism, hyperglycemia, and inflammation (Jones et al., 2021; Lu et al., 2022). While studies are limited, MR for methylation and cardiometabolic traits suggest that altered methylation is the consequence, not the cause, of metabolic dysfunction (Dekkers et al., 2016; Sayols-Baixeras et al., 2018; Zaghlool et al., 2018). It is possible that, in the setting of MetS, the catabolism of certain metabolites (e.g., serine) is altered and may induce methylation changes; and as such, DNA methylation functions as a biomarker of an existing metabolic disturbance. At the same time, the bidirectional influence of methylation on metabolic traits cannot be fully ruled out. For example, a nested case-control study found that methylation of cg06500161 at *ABCG1* was associated with incident diabetes in a population of adults in rural China (Qie et al., 2021). In our intermediate analyses of methylation and metabolites, we observed significant associations in the expected direction of effect based on prior studies, as well as a significant causal relationship of *ABCG1* methylation on serine. Thus, there may be biological plausibility for bidirectional relationships between the environment, metabolome, methylation, and MetS.

While this study was neither the first MWAS of MetS nor the MR of metabolites and methylation, it is one of the few to validate MetS-associated metabolites in an external cohort. And, to our knowledge, it is the first to evaluate bidirectional causality. In a multi-omics analysis, Zaghlool et al. detected a causal, inverse relationship of VLDL-A on cg00574958 at *CPT1A* (Zaghlool et al., 2018). In our findings, glucose was also inversely associated with this CpG site, whereas there was a positive causal association of serine. Furthermore, *ABCG1* methylation was causal on serine levels in reverse MR, whereas Zaghlool et al. were unable to investigate reverse causation. Overall, a lack of individual-level metabolite, methylation, and SNP data within a single cohort has limited investigators to one-sample and/or unidirectional MR approaches (Zaghlool et al., 2018; Wu et al., 2021). The former can lead to instrument bias, and in the latter, one cannot assess bidirectionality (Hartwig et al., 2016). We conducted MR with data from separate studies—thereby reducing the likelihood of instrument bias—and assessed reverse causation, unlike prior MR for DNA methylation and metabolites. Still, the number of participants in HyperGEN with available methylation and metabolite measurements were limited (N<150). This lack of power, coupled with the conservative Bonferroni-corrected significance threshold, may have led to false negatives. However, we also selected a group of metabolites with biological plausibility (i.e., potential methyl donors via DNA methyltransferase reactions) for causal testing even if they did not meet the corrected *p*-value threshold. Other limitations include between-study comparability, as REGARDS is a study of older adults (>45 years at baseline), and HyperGEN was enriched for hypertension. However, the robustness of our findings despite these cohort differences may suggest good generalizability.

In conclusion, we 1) conducted metabolite profiling for MetS; 2) evaluated the relationship between MetS metabolites and methylation of known MetS CpG sites; and 3) determined the causal direction of these relationships. Not only did we apply metabolite profiling in one of the largest and most diverse studies of MetS to date, but we also validated our findings in an external cohort for a total sample size of more than 2,000 individuals. The results showed that metabolites were causal on methylation, and one CpG-metabolite pair (cg00650161 (*ABCG1*)-serine) may also exhibit bidirectional influence. Our top findings suggested protective effects of serine in MetS, as well as causal relationships between serine and methylation of CpG sites linked to MetS. These findings were robust to multiple sensitivity analyses.

Longitudinal and mechanistic studies are needed to confirm these findings and further elucidate biological pathways. Further validation in more populations is also needed, as these relationships may vary in the context of different environments. Expanding our understanding of these relationships could potentially lead to the identification of precise biomarkers of MetS risk and cardiometabolic health overall.

## Data Availability

The data analyzed in this study is subject to the following licenses/restrictions: The metabolomics datasets used and/or analyzed during the current study are not publicly available but are available from the study upon reasonable request. However, genetic data and methylation data from HyperGEN can be accessed upon request via the TOPMed Consortium. Raw genotype data from REGARDS can be accessed via dbGaP (accession phs002719.v1.p1). Requests to access these datasets should be directed to REGARDS Study, regardsadmin@uab.edu; TOPMed Consortium, https://dbgap.ncbi.nlm.nih.gov/aa/wga.cgi?page&equals;login.
