# Supplementary material for: Metabolite profiles and DNA methylation in metabolic syndrome: a two-sample, bidirectional Mendelian randomization
Source: Front Genet. 2023 Sep 15;14:1184661. doi: 10.3389/fgene.2023.1184661 (PMC10540781; doi:10.3389/fgene.2023.1184661)
Supplement: Supplementary file 1 [file DataSheet1.ZIP › Jones_Supplement_Rev/Jones_Suppl_rev.docx]

**Metabolite profiles and DNA methylation in metabolic syndrome: a two-sample, bidirectional Mendelian randomization**

Supplementary Materials

Supplemental Figure 1: Summary of datasets utilized from REGARDS and HyperGEN studies

Supplemental Figure 2: Volcano plot of MetS metabolite associations in REGARDS discovery analysis

Supplemental Table 1: Summary statistics of metabolite profiling in REGARDS

Supplemental Table 2: Associations between prevalent MetS and metabolites selected for replication

Supplemental Table 3: Summary of reverse MR of CpG methylation on metabolites.

Supplemental Methods

S**upplemental Methods**

**REGARDS**

The Reasons for Geographical And Racial Differences in Stroke (REGARDS) Study is a prospective, national cohort study that was designed to observe racial and geographic differences in stroke incidence in the US, with oversampling in the Southeastern states with high stroke incidence. The cohort is composed of 30,239 white and black adults aged 45 and older. Eligibility screening and baseline data were collected via telephone survey and in-home physical assessment between 2003 and 2007. Follow-up data were collected an average of 10 years after enrollment. The REGARDS study design and objectives have been described elsewhere (1). The study protocol, as well as the ancillary studies for genomics and metabolomics, was approved by the institutional review boards (IRB) of all participating institutions, and all participants provided written informed consent. After the initial REGARDS data collection, blood samples from participants who consented to their data being used for ancillary studies, were further analyzed for genotyping and metabolomics analysis.

*Genotyping*: As part of an ancillary study on cardio-renal genomics, 10,903 REGARDS participants were selected for genotyping via Illumina Infinium Multi-Ethnic BeadChip (Extended MEGA-EX) array. DNA was extracted from whole blood samples collected at baseline. Genetic data were imputed using the National Heart, Lung, and Blood Institute (NHLBI) Trans-Omics in Precision Medicine (TOPMed) reference panel (Freeze 8). Participants were excluded if they had genotype call rates <95%, were internal duplicates, had sex mismatches, or were outliers (>6 standard deviations) on principal component analysis (PCA). After these quality control (QC) filters, there were 14,509,669 SNPs available for 10,643 participants.

*Metabolomics*: Within REGARDS, a case-cohort was created to evaluate risk factors for incident stroke among individuals without prevalent stroke (n=2,165) (2). Plasma samples were analyzed using targeted metabolomics as reported previously (3). Briefly, 30 uL aliquots of EDTA plasma were extracted using protein precipitation. Liquid chromatography-mass spectrometry (LC-MS/MS) was performed using dual Infinity II 1290 high-performance liquid chromatography pumps, Xbridge Amide columns (2.1 x 100 mm 3.5 μm; Waters), and a 6495 QQQ tandem mass spectrometer (Agilent) (4-6).

A total of 162 metabolites were detected, and all peaks were integrated and reviewed using MassHunter QQQ Quantitative Analysis software (Agilent). Following peak integration, each metabolite was normalized to the nearest pooled plasma samples, which were injected after every 10 study samples. Due to the nonparametric distribution of metabolite levels, all values underwent rank-based inverse normal transformation prior to statistical analyses. After exclusion of 126 participants missing phenotype data to ascertain MetS status, the final sample size of the metabolomics cohort was 2,039. Among these participants, 1,865 (91.5%) overlapped with the genomics cohort.

**HyperGEN**

The Hypertension Genetic Epidemiology Network (HyperGEN) is a cross-sectional, family-based study that is one of four networks of the NHLBI Family Blood Pressure Program designed to identify genetic risk factors for hypertension and hypertension-related end organ damage (7). The cohort includes sibships in which at least two siblings were diagnosed with hypertension before age 60, their unmedicated adult offspring, and age-matched controls for a total sample size of *n*~5000. Between 1995 and 2000, participants were recruited from Framingham, MA, USA; Minneapolis, MN, USA; Salt Lake City, UT, USA; Forsythe County, NC, USA; and Birmingham, AL, USA. All data were collected during in-person visit to the study field center. The study protocol was approved by the institutional review boards of all participating institutions, and all participants provided written informed consent.

*Genotyping*: Samples from participants who self-identified as Black or African American underwent whole-genome sequencing (WGS) through the NHLBI TOPMed program (N=1,898). We compiled a set of non-monomorphic and non-multiallelic SNPs with a minor allele frequency (MAF) > 1%. These filters yielded a total of 2,204,415 SNPs that were used as fence post markers for imputation using the same TOPMed release 2 reference panel as REGARDS.

*Epigenotyping*: As part of an ancillary study, 636 participants with echocardiography data were selected for epigenotyping to evaluate DNA methylation markers for left ventricular hypertrophy (8). Participants were selected using an extreme phenotype design, in which individuals from the highest and lowest quartiles of left ventricular mass were eligible for epigenotyping. DNA (500 ng) was isolated from buffy coat and hybridized to the Illumina Infinium HumanMethylation450 BeadChip (450K) array. Intensity files were analyzed using Illumina GenomeStudio, which generated cytosine-phosphate-guanine (CpG) beta (β) scores of the proportion of signaling of the methylation probe and corresponding p-value. QC filters removed CpG β scores with an association detection p-value >0.01, samples with >1.5% missing data points, and any CpG probe in which >10% of samples failed to yield adequate intensity. After these filters, there were 473,864 CpG sites eligible for analysis for 614 participants. Normalization was performed using the *SeSAMe* R package (“noob” function). Immune cell composition (CD8 T lymphocytes, CD4 T lymphocytes, natural killer (NK) cells, monocytes, and granulocytes) was estimated via the Houseman et al. algorithm (9). After exclusion of participants with missing phenotype data to ascertain MetS status, there were 557 participants who had both methylation and WGS data for analysis.

*Metabolomics*: Open profiling metabolomics analyses were conducted from plasma samples 300 African American participants. EDTA plasma from study participants age ≥40 years were assayed using an ultra-high performance liquid chromatography-tandem mass spectrometry (UPLC-MS/MS) platform from Metabolon (Durham, NC). The median relative standard deviation (RSD) for the instrument variability in the HyperGEN population was 4%, while the total process variability was 9%; both values met Metabolon’s acceptance criteria. Due to the nonparametric distribution of metabolite levels, all values underwent rank-based inverse normal transformation prior to statistical analyses. All participants had the necessary phenotype data to ascertain MetS status. There were 134 participants (44.7%) with available methylation data.

1. Howard VJ, Cushman M, Pulley L, Gomez CR, Go RC, Prineas RJ, et al. The reasons for geographic and racial differences in stroke study: objectives and design. Neuroepidemiology. 2005;25(3):135-43. Epub 2005/07/02. doi: 10.1159/000086678. PubMed PMID: 15990444.

2. Cushman M, Judd SE, Howard VJ, Kissela B, Gutiérrez OM, Jenny NS, et al. N-terminal pro-B-type natriuretic peptide and stroke risk: the reasons for geographic and racial differences in stroke cohort. Stroke. 2014;45(6):1646-50. Epub 20140422. doi: 10.1161/STROKEAHA.114.004712. PubMed PMID: 24757103; PubMed Central PMCID: PMC4142424.

3. Ament Z, Patki A, Chaudhary N, Bhave VM, Garcia Guarniz AL, Gao Y, et al. Nucleosides Associated With Incident Ischemic Stroke in the REGARDS and JHS Cohorts. Neurology. 2022;98(21):e2097-e107. Epub 20220309. doi: 10.1212/WNL.0000000000200262. PubMed PMID: 35264422; PubMed Central PMCID: PMC9169945.

4. Stapleton CJ, Acharjee A, Irvine HJ, Wolcott ZC, Patel AB, Kimberly WT. High-throughput metabolite profiling: identification of plasma taurine as a potential biomarker of functional outcome after aneurysmal subarachnoid hemorrhage. J Neurosurg. 2019:1-8. Epub 20191122. doi: 10.3171/2019.9.JNS191346. PubMed PMID: 31756713.

5. Ament Z, Bevers MB, Wolcott Z, Kimberly WT, Acharjee A. Uric Acid and Gluconic Acid as Predictors of Hyperglycemia and Cytotoxic Injury after Stroke. Transl Stroke Res. 2021;12(2):293-302. Epub 20201017. doi: 10.1007/s12975-020-00862-5. PubMed PMID: 33067777; PubMed Central PMCID: PMC7933067.

6. Kimberly WT, O'Sullivan JF, Nath AK, Keyes M, Shi X, Larson MG, et al. Metabolite profiling identifies anandamide as a biomarker of nonalcoholic steatohepatitis. JCI Insight. 2017;2(9). Epub 20170504. doi: 10.1172/jci.insight.92989. PubMed PMID: 28469090; PubMed Central PMCID: PMC5414569.

7. Williams RR, Rao DC, Ellison RC, Arnett DK, Heiss G, Oberman A, et al. NHLBI family blood pressure program: methodology and recruitment in the HyperGEN network. Hypertension genetic epidemiology network. Ann Epidemiol. 2000;10(6):389-400. Epub 2000/08/30. doi: 10.1016/s1047-2797(00)00063-6. PubMed PMID: 10964005.

8. Jones AC, Patki A, Claas SA, Tiwari HK, Chaudhary NS, Absher DM, et al. Differentially Methylated DNA Regions and Left Ventricular Hypertrophy in African Americans: A HyperGEN Study. Genes (Basel). 2022;13(10). Epub 20220922. doi: 10.3390/genes13101700. PubMed PMID: 36292585; PubMed Central PMCID: PMC9601679.

9. Houseman EA, Accomando WP, Koestler DC, Christensen BC, Marsit CJ, Nelson HH, et al. DNA methylation arrays as surrogate measures of cell mixture distribution. BMC Bioinformatics. 2012;13:86. Epub 2012/05/10. doi: 10.1186/1471-2105-13-86. PubMed PMID: 22568884; PubMed Central PMCID: PMC3532182.
