# Supplementary figures and images for: Metabolite profiles and DNA methylation in metabolic syndrome: a two-sample, bidirectional Mendelian randomization
Source: Front Genet. 2023 Sep 15;14:1184661. doi: 10.3389/fgene.2023.1184661 (PMC10540781; doi:10.3389/fgene.2023.1184661)

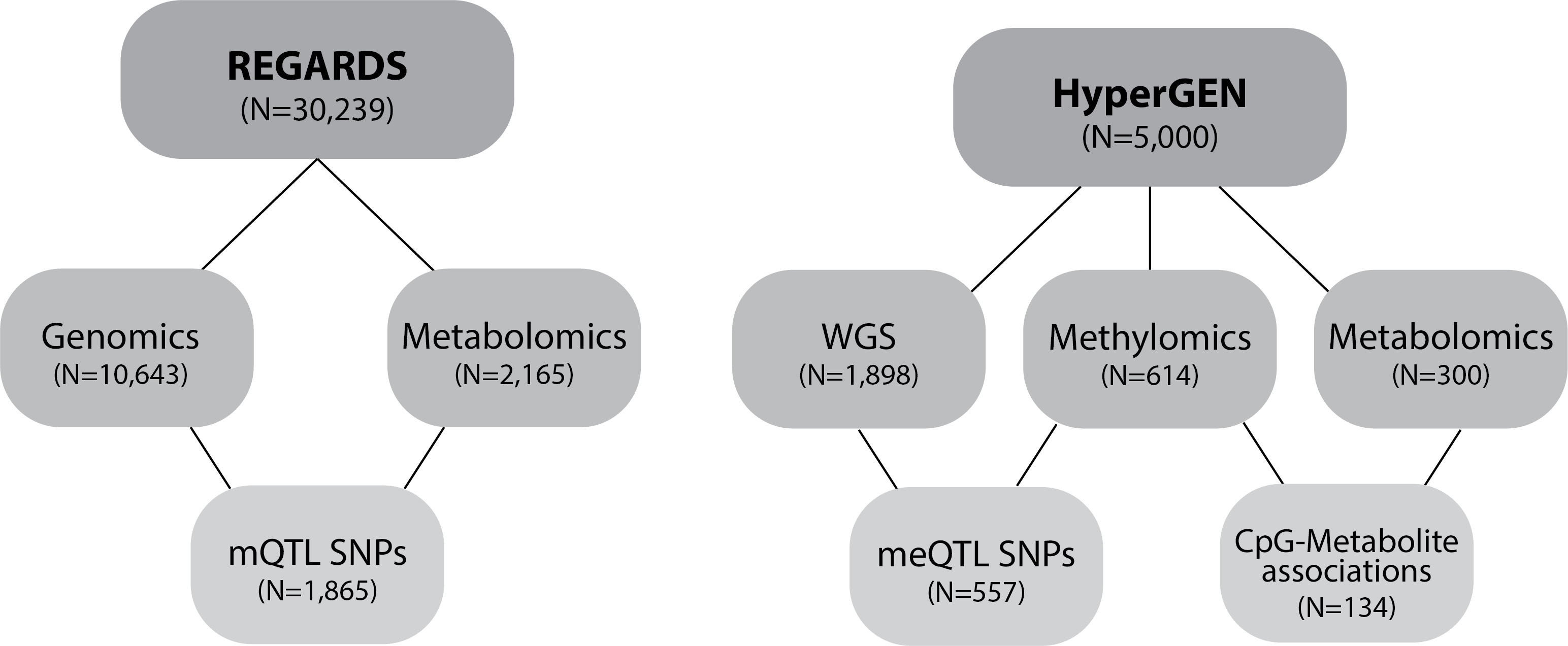

Supplement: Supplementary file 1 [file DataSheet1.ZIP › Jones_Supplement_Rev/Figure-S1.png]

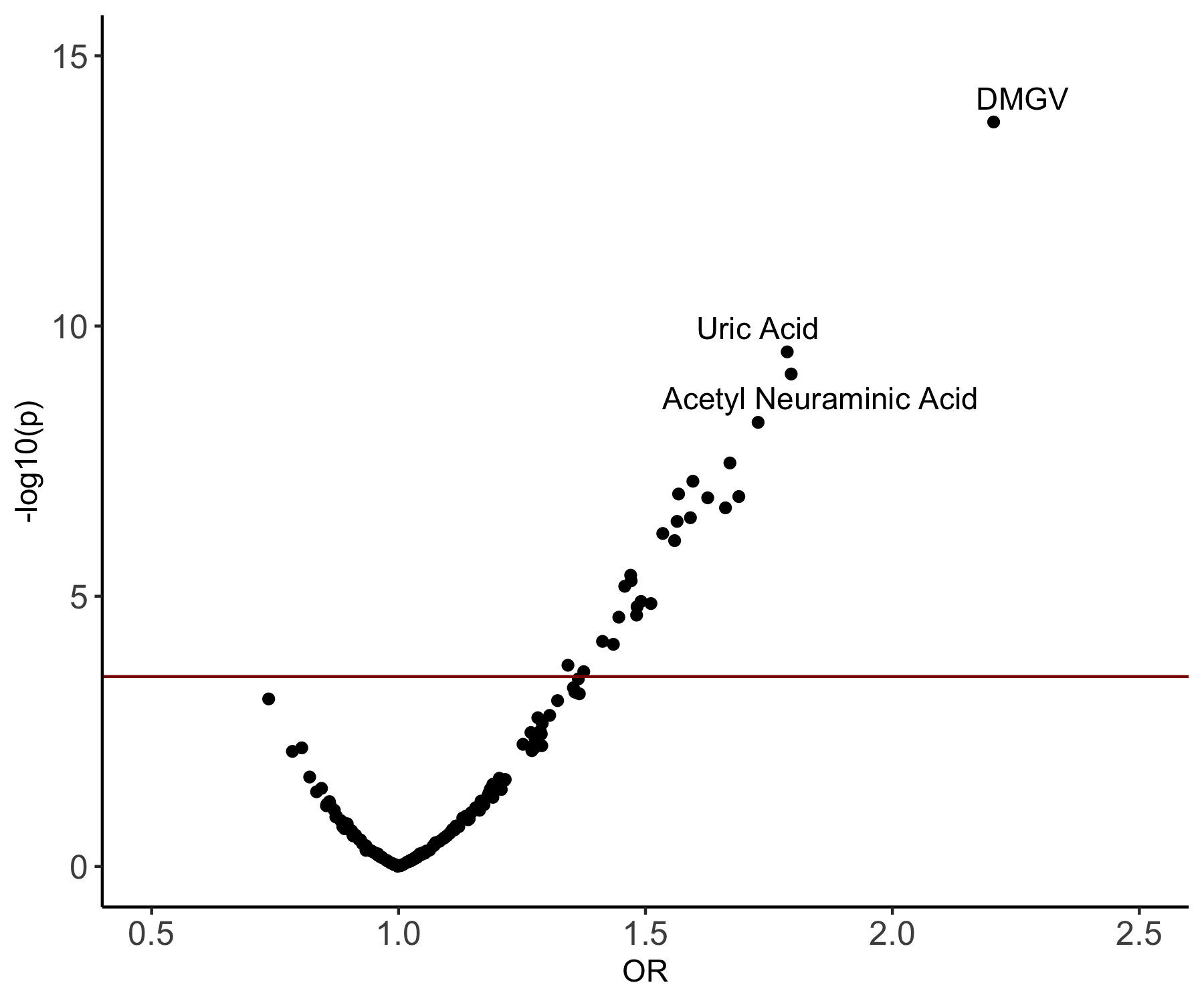

Supplement: Supplementary file 1 [file DataSheet1.ZIP › Jones_Supplement_Rev/Figure-S2.png]
